# Passing the Torch

**DOI:** 10.1371/journal.ppat.1008306

**Published:** 2020-01-30

**Authors:** Grant McFadden

**Affiliations:** Co-Editor-in-Chief of PLoS Pathogens

When I recently made the decision to step down as co-Editor-in-Chief of PLoS Pathogens, it gave me the opportunity to look back over the past 15 years since the first EIC of PP, John Young, asked me to join the PLoS journal as an Associate Editor. Back then, I couldn’t spell OA, let alone appreciate the seismic changes that the Open Access revolution would play in the communication of science. When the original founders of PLoS (Harold Varmus, Mike Eisen and Patrick Brown) proposed the new concept of the Creative Commons Attribution License back in 2004 that permitted the unrestricted use, distribution and reproduction of properly cited scientific work, the historical arc of scientific research publication was fundamentally inflected. What has transpired has included the good (wider access to scientific research by anyone with an internet connection), the bad (plagiarism, data manipulation) and the ugly (predatory journals, fake science). Regardless, there is no question that OA is now fundamentally key to the future of scientific publication.

For me, the realities of shrinking professional bandwidth forced me to conclude that I could no longer balance all of my other ever-growing duties with the time the PP co-EIC job deserves. The realities of shepherding a new biotech start-up company, OncoMyx, as well as trying to maintain funding for an independent, curiosity-based research lab in an era when the average PI needs to write 10 grant applications in order to get one funded, simply have come home to roost. But to balance my own angst of departing a journal that I have come to love dearly, I was more than thrilled to learn that my new replacement is Mike Malim, who is the perfect person to help lead the journal into the future. Welcome aboard, Mike!

There are a lot of people I want to thank from the PLoS family, both past and present. It takes a really big village to raise and sustain a journal like PP, a fact that is evident when you consider just the size of the PP Scientific Board of Editors: 65 Section Editors, 176 Associate Editors, 37 Frontmatter Editors, plus hundreds of invited Guest Editors over the years, all of which is managed by our core group of journal staff members. Of the many PP journal staffers I have worked with from the past, there are too many outstanding individuals to list but there are a few names I would never forgive myself for not including in a deserved shout-out: Catherine Nancarrow, Mary Kohut, Cory Mann, Laura Ray, Lily Berrin, and Sarah Sarber all made the day-to-day tasks of running the journal business a constant pleasure. The current PP staff roster continues that tradition of excellence, and Mike inherits a tremendous team that includes Eileen Clancy, Rebecca (Becs) Kirk, Claire Turner, and Rebecca Barilla. This is an exciting and transitional phase for all of the PLoS journals, and one of the things I will miss most is our regular meetings with the EICs of the other PLoS sister journals (you know who you are!) and with the newly constituted PLoS Board of Directors, led by Alison Mudditt. I especially want to make a very special thanks to Veronique Kiermer, Executive Editor, who continues to expertly steer the entire PLoS family from the past and into the future. Good show, Veronique! Finally, I want to thank my PP EIC partner-in-crime, Kasturi Haldar, for helping to oversee the journal with me for these past years. Kasturi, it has been an honor to work with you.

As numerous philosophers have opined (to be true to the spirit of OA, proper attribution requires at least a half dozen primary sources), it is difficult to make predictions, especially when they are about the future. But it is fair to say that as science evolves, scientific communication and publication must as well. In the case of PP, the focus of the journal has traditionally been on pathogenesis caused by identifiable pathogens, but perhaps future articles will give some much-deserved attention to commensals and microbiota that may not be directly pathogenic. When new areas emerge, PP has always been receptive to promoting them, and I suspect this will remain a core principle moving forward. Issues such as these are now in the hands of the new PP team, and I have no doubt that their decisions will be the right ones.

GMcF (Fig 1)

**Fig 1:**
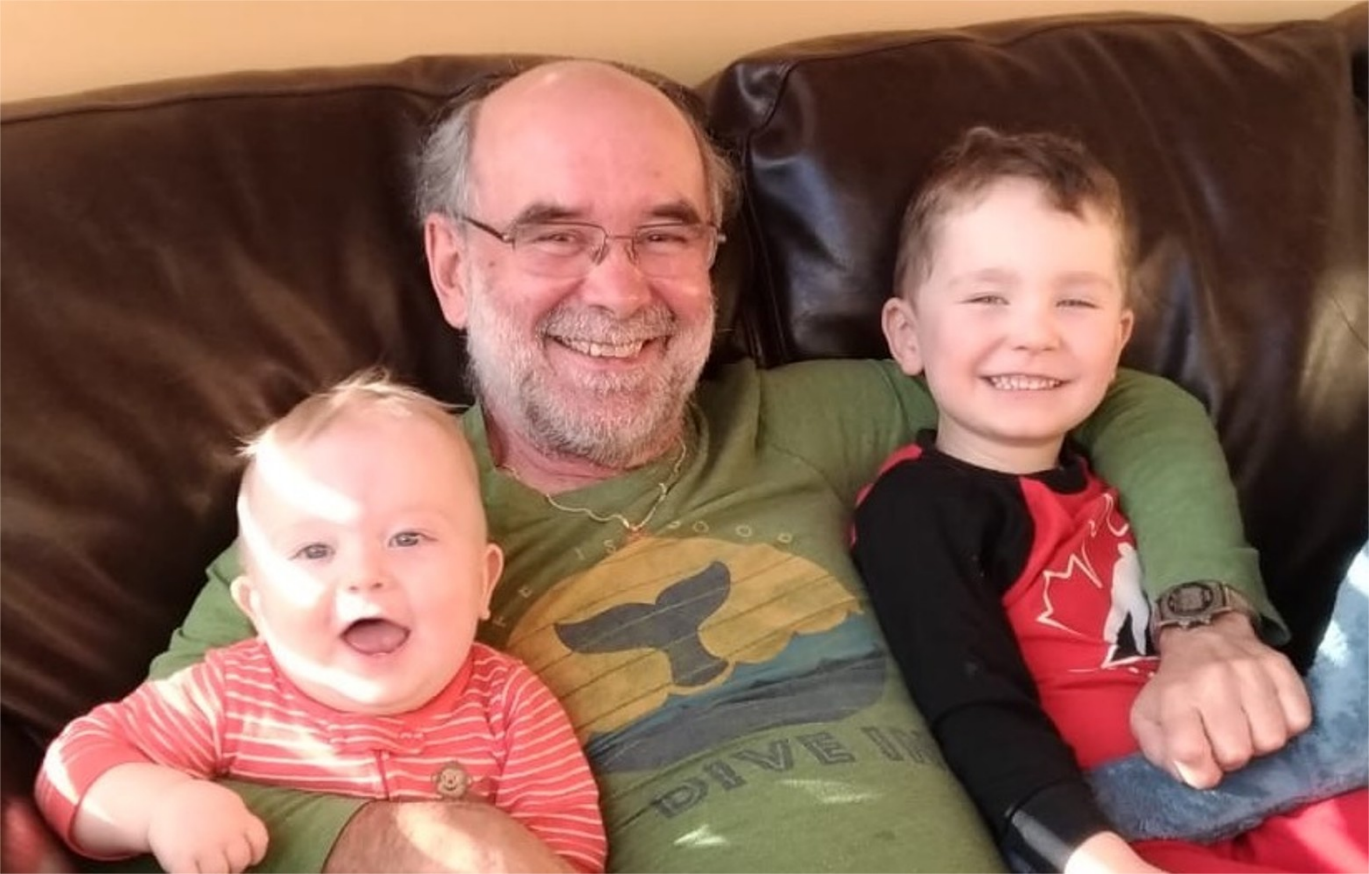
Grant McFadden, with his grandsons Aaron and Adam. Grant is the one in the middle.

December, 2019

